# Serum Metabolomics Study Reveals a Diagnostic Model for Lung Cancer Brain Metastasis

**DOI:** 10.1111/crj.70193

**Published:** 2026-05-05

**Authors:** Hongxia Zhu, Yinuo Jin, Xin You, Qi Wang

**Affiliations:** ^1^ Department of Respiratory Medicine the Second Affiliated Hospital of Dalian Medical University Dalian China; ^2^ Lung Cancer Transitional Medicine Center The Second Affiliated Hospital of Dalian Medical University Dalian China

## Abstract

Lung cancer remains the leading cause of cancer‐related deaths worldwide, with brain metastasis being one of the most common complications in advanced‐stage disease. The development of noninvasive and efficient early diagnostic methods is therefore of critical clinical importance. In this study, untargeted liquid chromatography–mass spectrometry (LC‐MS) was employed to perform metabolomic profiling of 66 serum samples from patients with lung cancer brain metastasis, early‐stage lung cancer, and healthy controls. A total of 719 metabolites were identified with high data reliability. Comparative analysis revealed 20 significantly upregulated and 12 significantly downregulated metabolites in the lung cancer brain metastasis group. These differentially expressed metabolites were primarily enriched in amino acid and energy metabolism pathways. This specific metabolic signature was highly associated with the brain metastatic state. Although not yet validated for clinical application, this profile demonstrated robust discriminatory power within the current cohort and serves as a potential set of risk‐stratification biomarkers. These findings identify a distinct metabolic phenotype associated with brain metastasis, laying the critical groundwork for future research into noninvasive diagnostic strategies. Nevertheless, further validation within independent, longitudinal cohorts is required.

## Graphical abstract

The overall workflow of noninvasive and efficient early diagnostic methods and key features of metabolic dysregulation associated with lung cancer brain metastasis, providing novel molecular insights into its pathogenesis and laying a foundation for future translational applications.

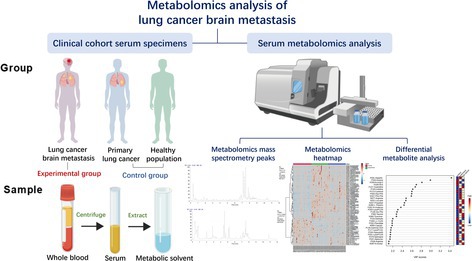

## 1 Introduction

According to the International Agency for Research on Cancer, nearly 20 million new cancer cases and 9.7 million deaths occurred worldwide in 2022, with lung cancer ranking first among all malignancies [1]. In China, the cancer burden is particularly severe, with 4.825 million new cases and over 2.574 million deaths in 2022 [2]. The brain is the most common metastatic site of lung cancer, significantly affecting neurological function and survival prognosis [3]. Brain metastasis occurs in 29% of non–small cell lung cancer (NSCLC) and 30% of small cell lung cancer (SCLC) cases [4]. These high incidence and mortality rates make lung cancer brain metastasis (LCBM) a considerable challenge in lung cancer management. Patients presenting with brain metastasis at initial diagnosis have better survival than those who develop it during disease progression, suggesting that early detection and timely systemic therapy can improve prognosis and increase survival rates in patients with LCBM [5]. However, because early stages of brain metastasis is often asymptomatic, neurological signs usually appear only at advanced stages, limiting treatment options and efficacy. Regular brain imaging monitoring (e.g., enhanced magnetic resonance imaging [MRI]) for high‐risk patients with lung cancer, combined with emerging technologies, such as liquid biopsy, may facilitate early detection of brain metastasis, timely intervention, prolonged survival, and improved quality of life.

Cancer development is closely linked to cellular metabolism. Tumor cells undergo “metabolic reprogramming” to sustain rapid proliferation and adapt to the tumor microenvironment, favoring glycolysis even under aerobic conditions (the “Warburg effect”), and enhancing glutamine metabolism, fatty acid synthesis, and nucleotide biosynthesis [6]. Alterations in these pathways are not merely downstream effects of genetic changes but also drive tumor growth, invasion, metastasis, and immune evasion through key signaling pathways [7]. Most metabolomics studies of lung cancer have focused on the metabolic characteristics of primary lesions. For example, abnormal regulatory networks involving nine metabolites and 57 metabolic genes have been identified in lung adenocarcinoma (LUAD) and lung squamous cell carcinoma (LUSC), with 28 genes associated with poor prognosis and six serving as independent prognostic markers [8]. Moreover, EGFR‐mutant LUAD enhances purine metabolism to sustain proliferation, highlighting the importance of metabolic reprogramming in lung cancer progression [9]. In our previous proteomic study of 26 paired lung cancer and adjacent normal tissues, we employed liquid chromatography–tandem mass spectrometry (LC‐MS/MS) with a data‐independent acquisition (DIA) mode and quantified 3152 proteins, including 189 upregulated and 522 downregulated proteins [10]. Furthermore, our team established a highly efficient platform for capturing circulating tumor cells (CTCs) and performed single‐cell metabolomic analyses, identifying 390 metabolites. CTCs with different metastatic tendencies exhibit characteristic “metabolic fingerprints.” Moreover, CTCs with a propensity for brain metastasis showed upregulation of phenylalanine metabolism, while those with a tendency for bone metastasis displayed activation of the arginine biosynthesis pathway [11].

Brain metastasis remains a critical determinant of lung cancer prognosis and occurs more frequently than metastases to other organs. However, systematic metabolomics analysis of LCBM and its microenvironment remains limited. The unique energy metabolism of brain tissue may drive specific metabolic adaptation mechanisms, with changes in metabolites often preceding morphological abnormalities. Metabolomics, as a key technology in biomarker research, demonstrates unique value in disease diagnosis and mechanism elucidation. Compared with genomics and proteomics, the metabolome positioned downstream in the biological information cascade more directly and sensitively reflects cellular function and real‐time physiological or pathological dynamics. Its high sensitivity enables detection of subtle early‐stage changes. Additionally, biofluid samples, such as serum and urine, can be obtained noninvasively, facilitating large‐scale screening and dynamic monitoring.

Therefore, the primary aim of this study is to elucidate the distinct serum metabolic signatures characteristic of LCBM and to identify potential noninvasive biomarkers for its early detection. To this end, we employed high‐throughput metabolomic profiling via LC‐MS on serum samples from patients with LCBM, early‐stage lung cancer, and healthy controls. We sought to construct a LCBM‐specific serum metabolic atlas and pinpoint the key regulatory nodes involved in metabolic reprogramming. Ultimately, this study aims to deepen the understanding of the metabolic driving mechanisms underlying LCBM and lay the foundation for developing highly sensitive blood‐based diagnostic tools, thereby facilitating early risk stratification and precision management of this disease.

## 2 Materials and Methods

### 2.1 Patient Selection

This study was approved by the Ethics Committee of the Second Affiliated Hospital of Dalian Medical University. Participants were recruited from patients treated at the Second Affiliated Hospital of Dalian Medical University between January 2023 and December 2024. Written informed consent was obtained from all participants.

According to internationally recognized diagnostic criteria [12], the study enrolled three distinct cohorts: patients with brain metastases from NSCLC, patients with primary NSCLC without brain metastases, and healthy controls. To ensure inter‐group comparability, the three cohorts were matched with respect to age, sex, and disease stage. Exclusion criteria included severe systemic diseases at the time of sample collection—such as renal or hepatic failure, acquired immunodeficiency syndrome (AIDS), inflammatory bowel disease, or systemic infections—as well as abnormal body mass index (BMI), either above or below the normal range. General clinical characteristics of the study groups are summarized in Table 1.

**TABLE 1:** Specimen information and statistical analysis of experimental and control groups.

		Lung cancer brain metastasis group	Primary lung cancer group	Healthy group	Variance	Chi‐square	*p*
Gender	Male	15	9	8	3.536		0.035*^a^
	Female	6	13	15			
Age	Average	63.62	61.91	59.65	0.999		0.374
	95% CI	60.24–67.00	58.74–65.07	54.33–64.98			
BMI	Average	24.13	23.64	24.55	0.373		0.690
	95% CI	22.70–25.56	21.94–25.34	23.02–26.08			
Tumor	0	0	0	—		15.831	0.001*
	1	6	19				
	2	5	2				
	3	4	1				
	4	6	0				
Node	0	0	15			33.302	0.001*
	1	2	6				
	2	12	1				
	3	7	0				
Metastasis	0	0	22			21.747	0.001*
	1	21	0				

*Note:* Study cohort and staging criteria: The study population was strictly limited to patients with NSCLC; SCLC was excluded. Detailed distributions of histological subtypes are presented in Table 2.

TNM staging for all patients was determined according to the 8th edition of the International Association for the Study of Lung Cancer (IASLC) staging system. The T (primary tumor) classification was based on tumor size and extent of local invasion: T1 (tumor ≤ 3 cm, subdivided into T1a [≤ 1 cm], T1b [> 1–2 cm], and T1c [> 2–3 cm]); T2 (tumor > 3 cm but ≤ 5 cm, or involvement of the main bronchus/visceral pleura, subdivided into T2a [> 3–4 cm] and T2b [> 4–5 cm]); T3 (tumor > 5 cm but ≤ 7 cm, separate tumor nodule(s) in the same lobe, or invasion of the chest wall/phrenic nerve/parietal pericardium); and T4 (tumor > 7 cm, separate tumor nodule(s) in a different ipsilateral lobe, or invasion of the mediastinum/diaphragm/heart/great vessels/recurrent laryngeal nerve). The N (regional lymph node) classification was based on the location of nodal metastasis: N0 (no regional lymph node metastasis); N1 (metastasis in ipsilateral peribronchial, hilar, or intrapulmonary nodes); N2 (metastasis in ipsilateral mediastinal or subcarinal nodes); and N3 (metastasis in contralateral mediastinal/hilar nodes, or any scalene/supraclavicular nodes). For M (distant metastasis), M0 indicated no distant metastasis, while M1 indicated the presence of distant metastasis; notably, all patients in LCBM group were classified as M1 with confirmed brain involvement. Given the significant intergroup differences observed in T, N, and M stages (all *p* < 0.001), which reflect distinct stages of disease progression, these variables were not matched between groups but were instead utilized as key labels characterizing disease status.

The asterisk (*) indicates a *p* value ≤ 0.05, representing a statistically significant difference. The superscript “a” in the table refers to the following note regarding Gender Distribution: Notably, a statistically significant difference in gender distribution was observed across the groups (*p* < 0.05), with a markedly higher proportion of male patients in the LCBM cohort. This finding suggests that male sex may serve as a potential high‐risk factor for brain metastasis within this population. The underlying mechanisms may be associated with androgen levels, cumulative smoking exposure, or other unmeasured sex‐specific biological characteristics. Consequently, sex was identified as a critical confounder and a potential effect modifier in this study. In subsequent predictive modeling and multivariable analyses, sex was explicitly included to adjust for its influence. Future studies are planned to further elucidate the specific regulatory roles of sex differences in metabolic profiling and susceptibility to brain metastasis.

Abbreviations: BMI, body mass index; NSCLC, non–small cell lung cancer; TNM, tumor–node–metastasis staging.

LCBM cohort was rigorously defined and characterized. All MRI scans were independently reviewed and validated by at least two senior neuroradiologists who were blinded to the patients' metabolic data. Diagnostic criteria adhered to the Response Assessment in Neuro‐Oncology (RANO) and RECIST 1.1 guidelines, requiring the presence of enhancing lesions consistent with metastatic disease in the context of a known primary NSCLC. Cases with ambiguous imaging findings where primary brain tumors (e.g., gliomas) or nonneoplastic lesions (e.g., abscesses) could not be excluded via pathology were excluded. To characterize the disease burden within the LCBM cohort, detailed metrics were recorded for the experimental group, including the number of metastases (categorized as solitary vs. multiple) and the maximum diameter of the largest lesion; these characteristics are summarized in the Tables 1 and 2.

**TABLE 2:** Baseline characteristics and brain metastasis profiles of patients with lung cancer brain metastasis (LCBM).

LCBM group	Sex	Smoking status	Pack‐year	Primary histology	Brain metastasis (location–size, mm)	Oncogene dependency status
No. 1	Male	Never	0	Adeno	L‐Frontal (11)	EGFR L858R
No. 2	Male	Current	40	Adeno	Mult. (7)	—
No. 3	Male	Former	40	Adeno	L‐Parietal (3)	—
No. 4	Female	Never	0	Adeno	L‐Thal (7)	—
No. 5	Female	Never	0	Adeno	L‐Vent (5)	EGFR L858R
No. 6	Female	Never	0	Adeno	R‐Frontal (4)	TP53
No. 7	Male	Former	10	Adeno	R‐Vent (7)	—
No. 8	Female	Never	0	Adeno	R‐Parietal (3)	EGFR T790M
No. 9	Male	Current	40	Adeno	L‐Frontal (4)	EGFR 19del
No. 10	Female	Never	0	Adeno	R‐Vent (8)	EGFR L858R
No. 11	Male	Former	20	Adeno	L‐Vent (3)	EGFR T790M
No. 12	Male	Current	20	Adeno	R‐Vent (3)	TP53
No. 13	Male	Former	30	SqCC	R‐Temporal (6)	—
No. 14	Male	Current	40	SqCC	CR (3)	KRAS
No. 15	Female	Never	0	Adeno	Mult. (8)	—
No. 16	Male	Current	40	SqCC	Bilat. Temporal (38 × 33)	—
No. 17	Male	Current	40	Adeno	L‐Frontal (2)	TP53
No. 18	Male	Former	20	Adeno	R‐Occipital (4)	—
No. 19	Male	Former	50	SqCC	Mult. (31 × 29)	—
No. 20	Male	Former	3	Adeno	L‐Parietal (8)	—
No. 21	Male	Current	30	Adeno	Mult. (25 × 20)	ALK fusion

*Note:* Lesion size: Dimensions are reported as the maximum diameter in millimeters (mm). For elliptical lesions (e.g., 38 × 33 mm), the larger value represents the maximum diameter. Multiple Lesions: “Mult.” indicates the presence of multiple metastatic nodules where individual counts were not specified in the initial imaging report; the value in parentheses represents the size of the dominant or largest lesion.

Oncogene status: Specific driver mutations (e.g., EGFR L858R, EGFR exon 19 deletion, ALK rearrangement) are listed. A hyphen (−) indicates wild‐type status for the tested panel or that comprehensive molecular profiling was not performed/available.

Smoking status: “Never” smokers (< 100 cigarettes in lifetime); “Former” smokers (quit > 1 year prior to diagnosis); “Current” smokers.

Abbreviations: Adeno, adenocarcinoma; CR, corona radiata; Frontal/Parietal/Temporal/Occip., frontal/parietal/temporal/occipital lobe; L/R/Bilat., left/right/bilateral; Mult., multiple lesions (≥ 2 nodules); Pack‐Year, cumulative tobacco exposure calculated as packs per day multiplied by years smoked; SqCC, squamous cell carcinoma; Thal, thalamus; Vent, ventricle.

The primary NSCLC control group was strictly defined as patients with primary NSCLC with no evidence of distant metastasis. This cohort encompassed various pathological stages, predominantly Stage I and II, including adenocarcinoma in situ (AIS), minimally invasive adenocarcinoma (MIA), and invasive adenocarcinoma or squamous cell carcinoma, reflecting their clinical status prior to the development of distant metastases. The detailed distribution of pathological subtypes and staging for this group is provided in the Table 3.

**TABLE 3:** Demographic, clinical, and pathological characteristics of patients with early‐stage lung cancer (*N* = 22).

Early‐stage group	Sex	Smoking status	Pack‐years	Lesion characteristics (location and size)	Primary histology	Treatment
No. 1	Male	Former	10	RLL, 41 mm	Inv. Adeno	Surgery
No. 2	Male	Current	50	RUL, 11 mm	SqCC	Neoadjuvant Therapy
No. 3	Male	Never	0	RUL, 8 mm	AIS	Surgery
No. 4	Female	Never	0	LLL, 9 mm	AIS	Surgery
No. 5	Male	Never	0	LLL, 28 mm	Inv. Adeno	Surgery
No. 6	Female	Never	0	RUL, 11 mm	Inv. Adeno	Surgery
No. 7	Female	Never	0	RUL, 32 mm	Inv. Adeno	Surgery
No. 8	Female	Never	0	RLL, 15 mm	MIA	Surgery
No. 9	Male	Never	0	RML, 18 mm	Inv. Adeno	Surgery
No. 10	Female	Never	0	RUL, 9 mm	Inv. Adeno	Surgery
No. 11	Male	Never	0	RML, 16 mm	Inv. Adeno	Adjuvant Therapy
No. 12	Female	Never	0	RLL, 66 mm	Inv. Adeno	Surgery
No. 13	Female	Never	0	RUL, 61 mm	Inv. Adeno	Surgery
No. 14	Female	Never	0	RUL, 23 mm	Inv. Adeno	Surgery
No. 15	Male	Never	0	RUL, 30 mm	Inv. Adeno	Surgery
No. 16	Female	Former	40	LLL, 27 mm	Inv. Adeno	Adjuvant Therapy
No. 17	Male	Current	60	RUL, 26 mm	Inv. Adeno	Surgery
No. 18	Female	Never	0	RML, 12 mm	MIA	Surgery
No. 19	Male	Never	0	LLL, 18 mm	Inv. Adeno	Surgery
No. 20	Female	Never	0	RUL, 15 mm	Inv. Adeno	Surgery
No. 21	Female	Never	0	RUL, 9 mm	AIS	Surgery
No. 22	Female	Never	0	LLL, 15 mm	MIA	Surgery

*Note:* Lesion characteristics: Tumor location is indicated by lung lobe abbreviation. Size represents the maximum diameter of the solid or ground‐glass component in millimeters (mm) as measured on preoperative high‐resolution computed tomography (HRCT).

Histological classification: Pathological diagnoses were confirmed post‐surgery according to the WHO Classification of Thoracic Tumors (5th edition). AIS and MIA represent pre‐invasive and minimally invasive stages, respectively, while “Inv. Adeno” denotes frankly invasive adenocarcinoma.

Surgery: Primary treatment consisting of anatomical resection (lobectomy or segmentectomy) with systematic lymph node dissection.

Neoadjuvant therapy: Systemic treatment (chemotherapy and/or immunotherapy) administered prior to surgical resection.

Adjuvant therapy: Postoperative systemic treatment administered based on pathological staging and risk stratification.

Smoking history: Categorized as Never (< 100 cigarettes in lifetime), Former (ceased > 1 year prior to diagnosis), or Current smoker. Pack‐years = (packs smoked per day) × (years of smoking).

Abbreviations: AIS, adenocarcinoma in situ; Inv. Adeno, invasive adenocarcinoma; LLL, left lower lobe; MIA, minimally invasive adenocarcinoma; RLL, right lower lobe; RML, right middle lobe; RUL, right upper lobe; SqCC, squamous cell carcinoma.

To effectively address the significant molecular heterogeneity of NSCLC, our retrospective cohort construction not only incorporated initial clinical symptoms and imaging features as enrollment criteria but also systematically integrated driver gene status as a key stratification variable. The study cohort included a substantial proportion of oncogene‐addicted cases, with the EGFR mutation subgroup being particularly prominent. Guided by confirmed molecular pathology results, we conducted an in‐depth analysis in the discussion section to elucidate the intrinsic association between specific oncogenic driving mechanisms—particularly the EGFR signaling pathway—and serum metabolic profiles, thereby revealing unique metabolic reprogramming patterns across different molecular subtypes.

### 2.2 Sample Collection and Storage

To ensure that the identified metabolic signatures reflected the intrinsic disease state rather than therapeutic effects, strict criteria regarding sampling timing were implemented. All blood samples were collected during hospitalization, specifically at the time of initial admission and diagnosis, prior to the initiation of any specific anti‐tumor therapies (including chemotherapy, radiotherapy, targeted therapy, or immunotherapy).

Adhering to ethical principles aimed at minimizing patient burden, no additional venipunctures were performed solely for research purposes. Approximately 5 mL of blood was drawn from each participant into plain vacuum tubes without anticoagulants. Samples were allowed to clot at room temperature for 30–60 min. Subsequently, clotted blood was centrifuged at 3000 × g for 20 min at 4°C to separate the serum. The supernatant (serum) was carefully aspirated, avoiding the buffy coat to prevent cellular contamination.

Serum separation was completed within 4 h of blood collection. Serum aliquots were immediately transferred to sterile cryovials and stored at −80°C until analysis. Strict protocols were followed to limit freeze–thaw cycles; all samples underwent only a single freeze–thaw cycle (thawed on ice immediately prior to metabolomic analysis) to prevent the degradation of labile metabolites. No anticoagulants (e.g., EDTA, heparin, or citrate) were used, as these are specific to serum preparation and may interfere with metabolic profiles. The uniform use of serum and standardized processing conditions across the LCBM, early‐stage lung cancer, and healthy control groups ensured that observed metabolic differences were attributable to disease status rather than pre‐analytical artifacts.

### 2.3 Chemicals and Reagents

Lysophosphatidylcholine C19:0 (LPC 19:0) was purchased from Avanti Polar Lipids (Alabaster, AL, USA); Phe‐d5, Trp‐d5, FA 18:0‐d3, FA 16:0‐d3, carnitine C16:0‐d3, formic acid (FA), and ammonium bicarbonate were obtained from Sigma (St. Louis, MO, USA); CDCA‐d4 and CA‐d4 were purchased from TRC (Canada); acetonitrile (ACN) and methanol were purchased from Merck (Darmstadt, Germany); and pure water was prepared using a Milli‐Q purification system (Millipore, Milford, MA, USA).

### 2.4 Sample Preparation

Each serum sample (50 μL) was mixed with 200 μL of internal standard solution (LPC 19:0 3 μg/mL, FA 18:0‐d3 2.5 μg/mL, FA 16:0‐d3 2.5 μg/mL, Phe‐d5 3.6 μg/mL, Trp‐d5 4.25 μg/mL, CDCA‐d4 1.5 μg/mL, CA‐d4 1.85 μg/mL, Carnitine C2:0‐d3 0.16 μg/mL, Carnitine C8:0‐d3 0.1 μg/mL, Carnitine C16:0‐d3 0.15 μg/mL in methanol), and vortexed for 2 min. The mixed samples were then centrifuged for 4 min (20 000 × g, 4°C). A 180‐μL aliquot of the resulting supernatant was lyophilized and stored at −80°C until further use. The remaining supernatant was pooled and processed identically to generate quality control (QC) samples.

Before mass spectrometry (MS) analysis, 50 μL of 25% ACN solution was added to each sample (including QC samples), vortexed for 2 min (4°C, 1800 rpm), and centrifuged for 10 min (20 000 × g, 4°C). The supernatant was collected for MS analysis.

### 2.5 MS Analysis

Serum samples were analyzed using a Q‐Exactive mass spectrometer equipped with a nanospray ion source and a Vanquish UHPLC system (Thermo Fisher Scientific, San Jose, CA, USA).

### 2.6 HPLC Conditions

#### 2.6.1 Positive Ion Mode

The analytical column was packed with C8 beads (50 mm × 2.1 mm, 1.7 μm, Waters, Milford, MA). Column temperature was set at 60°C, and flowrate at 0.4 mL/min. The gradient was as follows: 0–0.5 min, 5% Phase B (100% ACN/0.1% FA) and 95% Phase A (100% H2O/0.1% FA); 0.5–1.5 min, 5%–40% Phase B; 1.5–6 min, 40%–100% Phase B; 6–8 min, 100% Phase B; 10–10.1 min, 100%–5% Phase B; 10.1–12 min, 5% Phase B.

#### 2.6.2 Negative Ion Mode

The analytical column was ACQUITY UPLC HSS T3 (50 mm × 2.1 mm, 1.8 μm; Waters, Milford, MA, USA). Column temperature was set at 60°C, and flowrate at 0.4 mL/min. The gradient was as follows: 0–0.5 min, 2% Phase B (6.5 mM NH4HCO3 in 95% methanol/5% H2O) and 98% Phase A (6.5 mM NH4HCO3 in 100% H2O); 0.5–2 min, 2%–40% Phase B; 2–8 min, 40%–100% Phase B; 8–10 min, 100% Phase B; 10–10.1 min, 100%–2% Phase B; and 10.1–12 min, 2% Phase B.

#### 2.6.3 MS Conditions

The electrospray voltage of 3.5 kV (ESI+) and 3.0 kV (ESI‐), with a capillary column temperature of 320°C versus the inlet of the mass spectrometer, was used. The full scan mode was employed with scan range *m*/*z* = 70–1050 and resolution of 14e4.

### 2.7 Quality Control

To monitor analytical stability, one QC sample was analyzed after every 10 serum samples. QC samples were prepared by pooling equal volumes of all serum supernatants.

## 3 Results and Discussion

**FIGURE 1:**
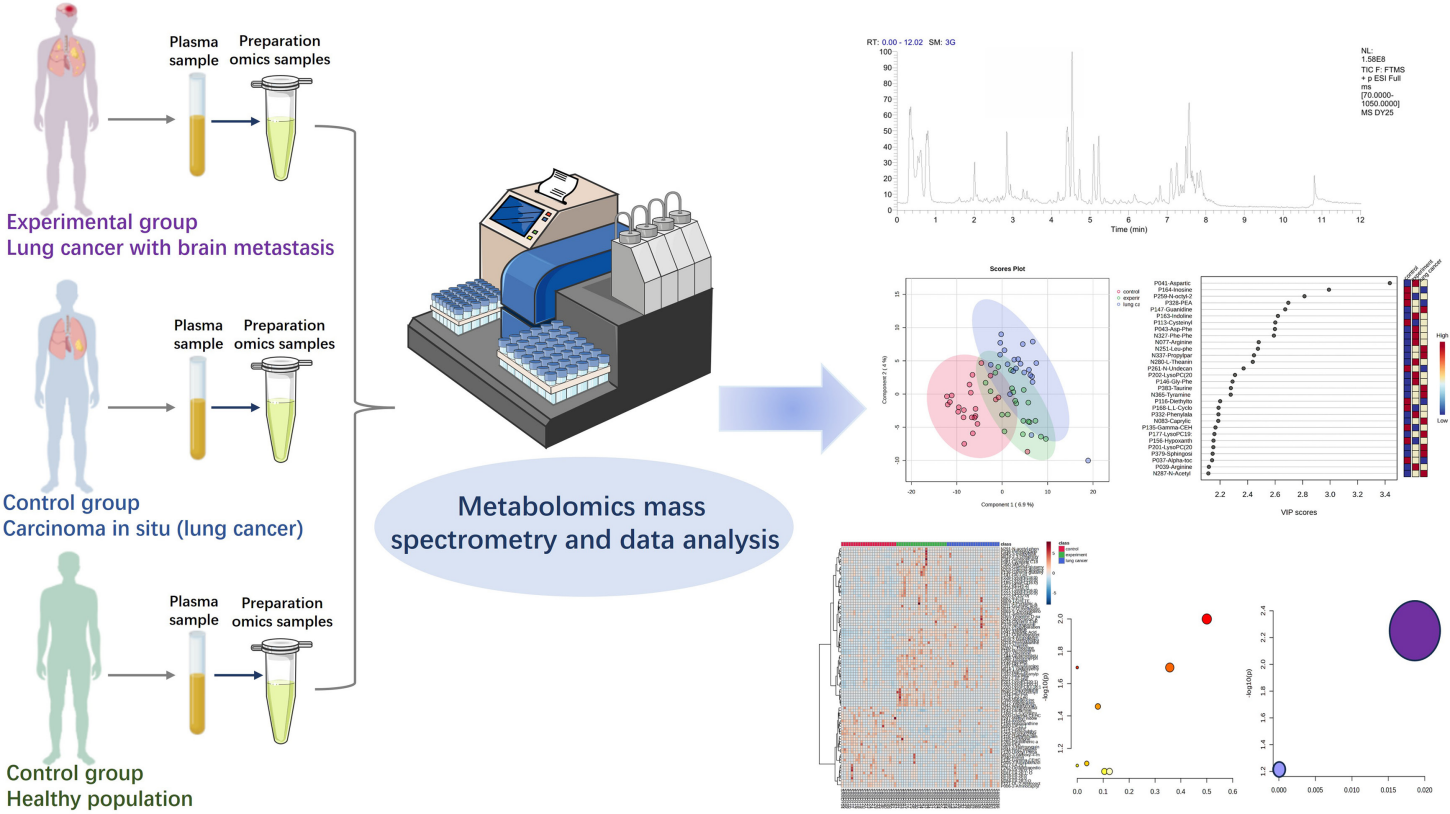
Workflow diagram of serum metabolomics study in lung cancer brain metastasis.

The overall workflow of this study is illustrated in Figure 1. LC‐MS was employed to systematically analyze the serum metabolites of patients with LCBM, early‐stage lung cancer, and healthy controls. A total of 66 serum samples were included: 21 from patients with LCBM, 22 from early‐stage lung cancer patients, and 23 from healthy controls. Untargeted metabolomic profiling was performed using a Thermo Scientific Vanquish UHPLC system coupled with a Q Exactive mass spectrometer, with a QC sample analyzed after every 10 runs to monitor analytical stability; detailed mass spectrometry data can be found in Table S1.

**FIGURE 2:**
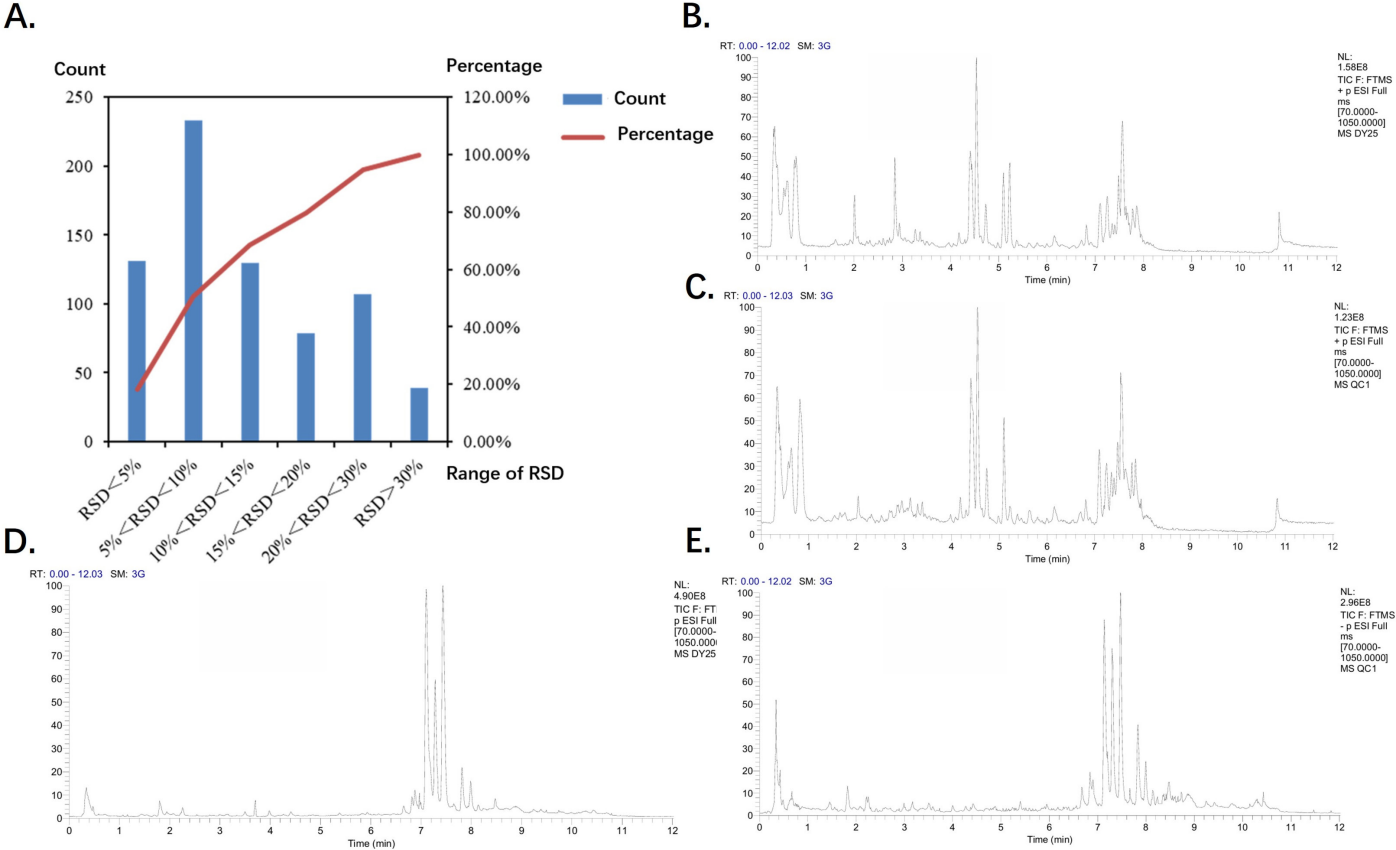
Quality control (QC) and sample total ion chromatograms (TIC) in positive and negative ion modes, and distribution of relative standard deviation (RSD) for metabolite components. This figure illustrates the TIC of QC and experimental samples in both positive and negative ion modes, along with the distribution of RSD for metabolite components in QC samples. It is used to assess system stability and data reliability. (A) Distribution of RSD for metabolite components in QC samples; (B) sample TIC chart in positive ion mode; (C) TIC chart of QC samples in positive ion mode; (D) TIC chart of samples in negative ion mode; (E) TIC chart of QC samples in negative ion mode.

In total, 719 metabolites were identified, with only eight exhibiting a relative standard deviation (RSD) exceeding 30% in QC samples (1.65%), indicating excellent reproducibility and compliance with metabolomics quality standards (Figure 2). This high‐quality dataset provides a robust foundation for subsequent identification of metabolic pathway alterations and potential biomarkers associated with LCBM, offering insight into its underlying metabolic reprogramming mechanisms.

**FIGURE 3:**
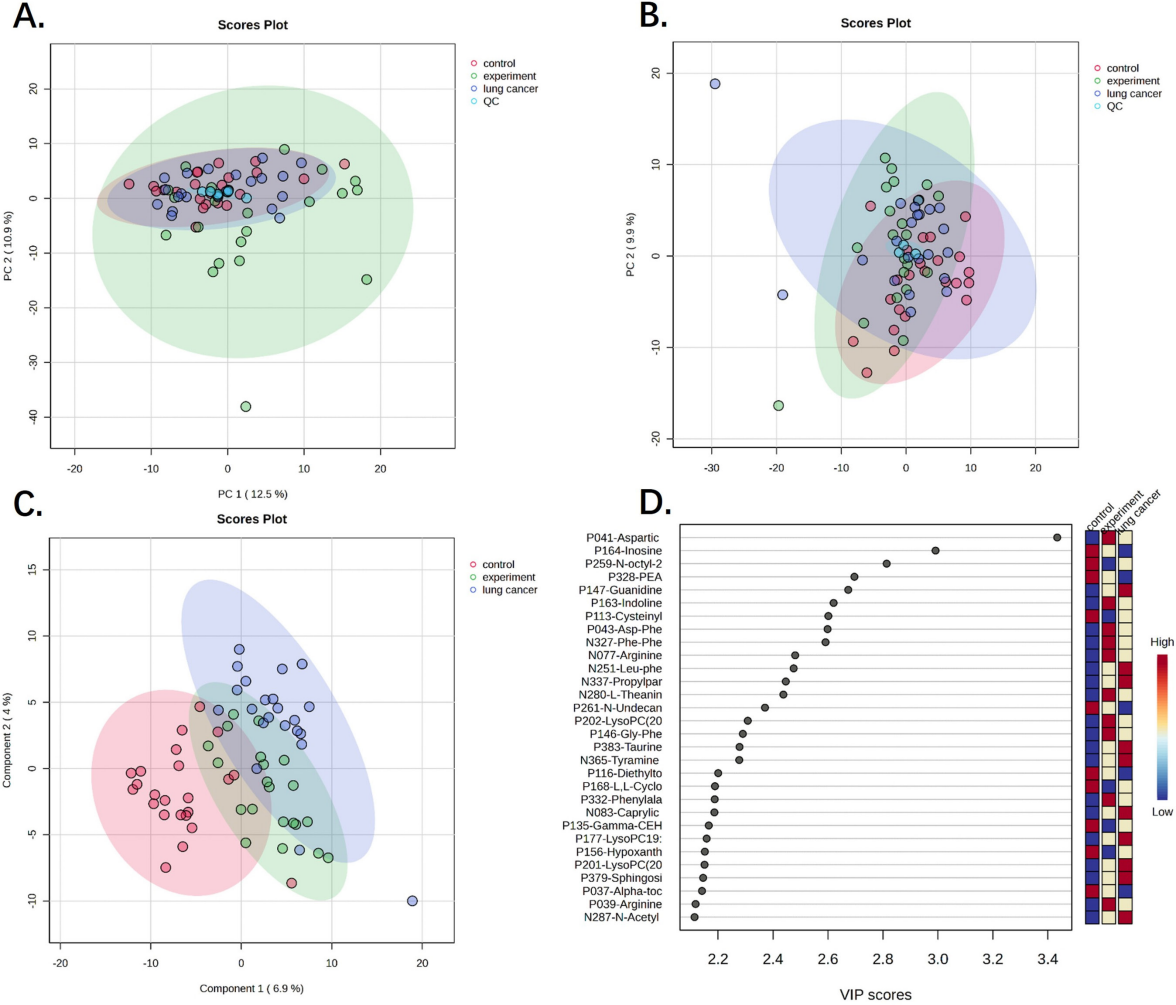
PCA and PLS‐DA Score plots in positive and negative ion modes, and analysis of the top 30 metabolites by VIP value. The figure presents PCA and PLS‐DA score plots illustrating metabolic differences between groups in both ion modes and highlights the top 30 potential biomarkers with the highest VIP values contributing to group separation. (A) PCA score plot in positive ion mode; (B) PCA score plot in negative ion mode; (C) PLS‐DA score plot of all components from experimental, lung cancer and control groups; (D) Top 30 metabolites by VIP value.

**FIGURE 4:**
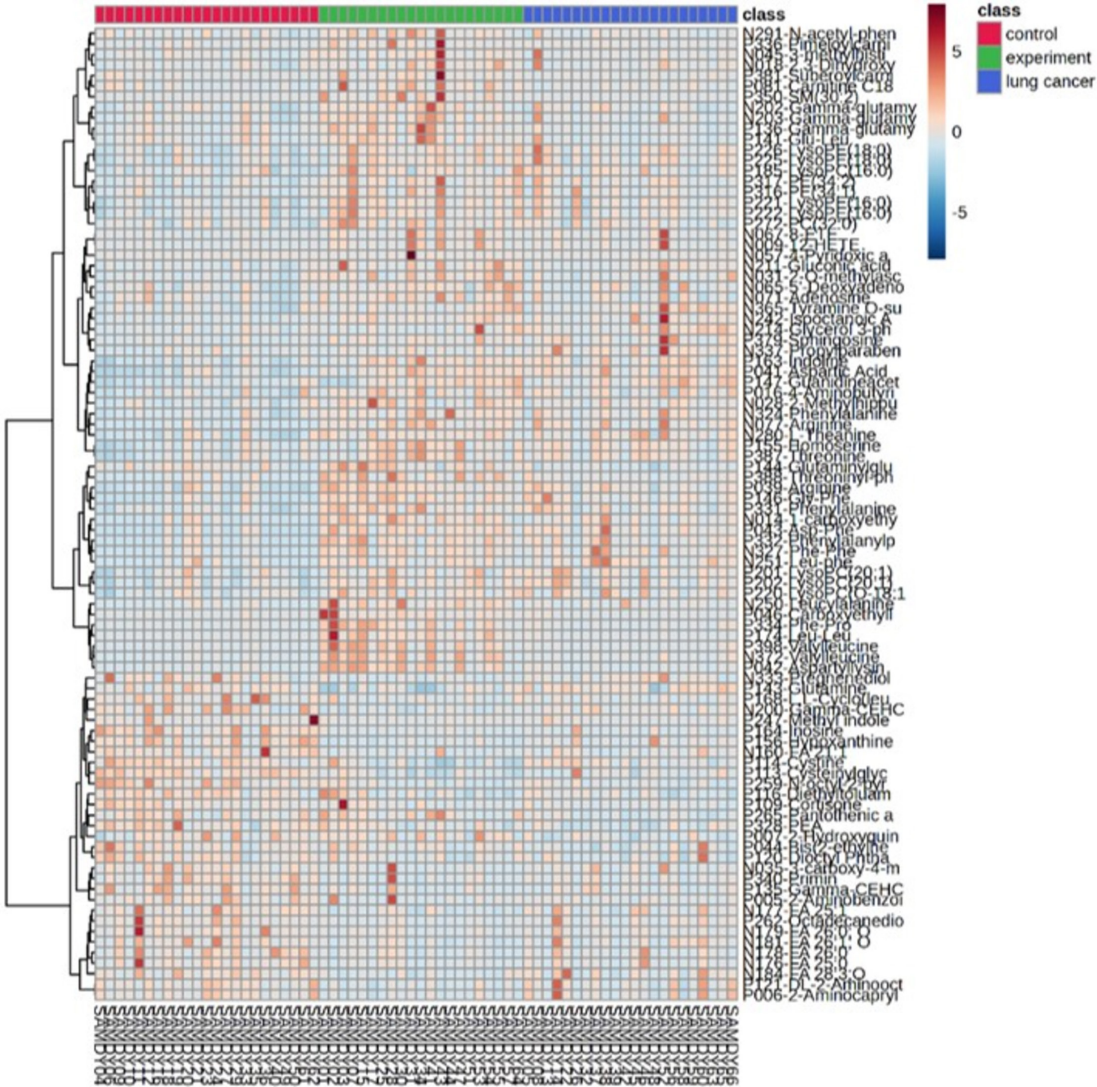
Heatmap of differentially metabolites selected by ANOVA. Clustered heatmap of serum metabolites differentially expressed in lung cancer brain metastasis, selected by ANOVA.

Based on the metabolomics analysis results of serum samples and using principal component analysis (PCA), Figure 3 preliminarily and intuitively classifies the metabolic outcomes of the three patient groups, highlighting the overall metabolomic differences between groups and the variability within groups. Comprehensive analysis of serum samples identified 719 metabolites. Multivariate (PCA and PLS‐DA) and univariate (ANOVA) analyses were performed on these metabolites across the three sample groups. The PLS‐DA results are provided in Table S2, and the ANOVA results are presented in Table S3. Differentially expressed metabolites identified according to the screening criteria (top 30 metabolites ranked by VIP value and *p* < 0.05) are presented in Figure 4. In the LCBM group, 20 metabolites were significantly upregulated, mainly enriched in phenylalanine, arginine, serine, and threonine metabolism pathways, whereas 12 metabolites were significantly downregulated, primarily related to cysteine, methionine, and purine metabolism pathways. Previous studies on the metabolomic profiles of NSCLC with brain metastasis identified a diagnostic biomarker panel comprising cysteine, ascorbic acid, LPC (22:0), and LPC (20:0), capable of distinguishing brain metastases from primary tumors [13]. This finding aligns with the trend observed for some metabolites in our study.

**FIGURE 5:**
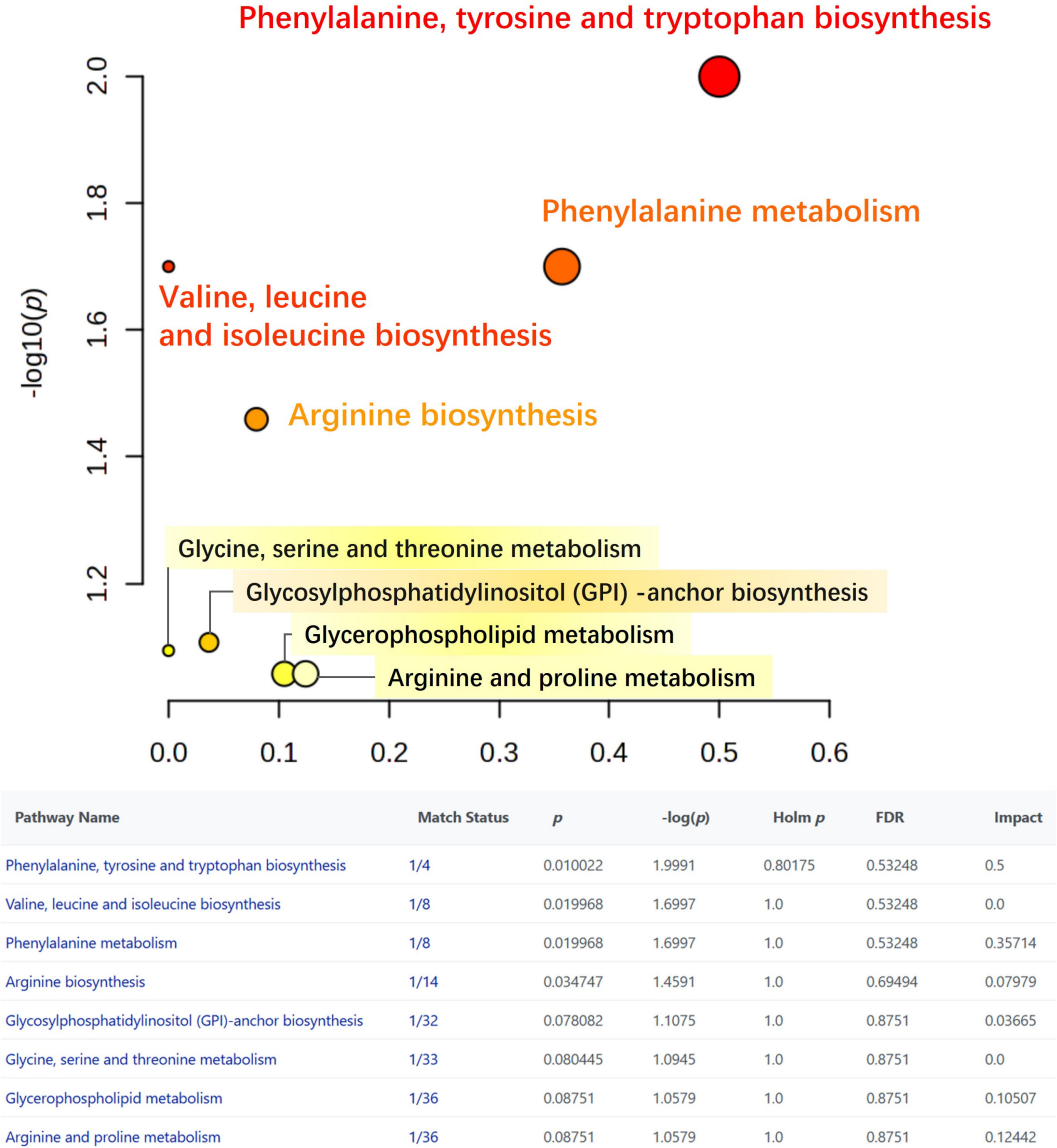
Overview of differential elevated metabolite pathway analysis. KEGG pathway enrichment analysis was performed on differentially upregulated metabolites, with the highest correlation observed in the phenylalanine metabolism pathway, followed by the valine and arginine metabolism pathways.

Pathway enrichment analysis (Figure 5) demonstrated that the upregulated metabolites—predominantly phenylalanine, arginine, serine, and threonine—were closely associated with tumor progression, suggesting their potential as biomarkers for disease progression. Recent studies further support this observation. Li et al. reported that phenylalanine hydroxylase–mediated phenylalanine metabolism activates the mTORC1 signaling pathway, promoting glycolysis and drug efflux, thereby contributing to chemotherapy resistance in bladder cancer [14]. Similarly, Gao et al. found that phenylalanine enhances NSCLC cell proliferation, migration, and invasion, indicating its potential as a predictive factor for tumor metastasis [15]. Additionally, metabolomic profiles from liquid biopsies have been linked to survival outcomes in patients receiving immune checkpoint inhibitors. For instance, metabolites such as alanine, aspartate, glutathione, histidine, isoleucine, and phenylalanine were significantly associated with survival in patients treated with ipilimumab [16]. Our group has previously reported, through single‐cell metabolic analysis of circulating tumor cells, that multiple metabolites, including phenylalanine, hold potential as therapeutic targets and prognostic markers [11]. Mechanistically, phenylalanine participates in several key metabolic pathways. In tumor cells, phenylalanine can be converted into tyrosine, which subsequently contributes to the synthesis of neurotransmitters and hormones, such as dopamine and epinephrine [17]. In addition, phenylalanine enters the tricarboxylic acid (TCA) cycle through oxidative decomposition, providing energy and biosynthetic precursors essential for tumor cell survival and proliferation [18]. Phenylalanine also promotes lipid synthesis, thereby supporting tumor growth and metabolic activity [19]. Moreover, it interacts with endoplasmic reticulum proteins to activate the PERK–ELF2A pathway, leading to apoptosis [20] and induces ATF3 expression, which subsequently activates CHOP and DR5 to promote apoptosis [21]. In our study, phenylalanine levels were elevated in the serum of patients with lung cancer and further increased in those with high tumor burden in brain metastasis, indicating that phenylalanine may reflect tumor metabolic reprogramming and serves as a potential biomarker. Future studies should investigate the specific mechanisms by which phenylalanine contributes to LCBM. A deeper understanding of phenylalanine and its associated metabolic pathways could facilitate the development of more precise and effective therapeutic strategies and prognostic tools for patients with LCBM.

**FIGURE 6:**
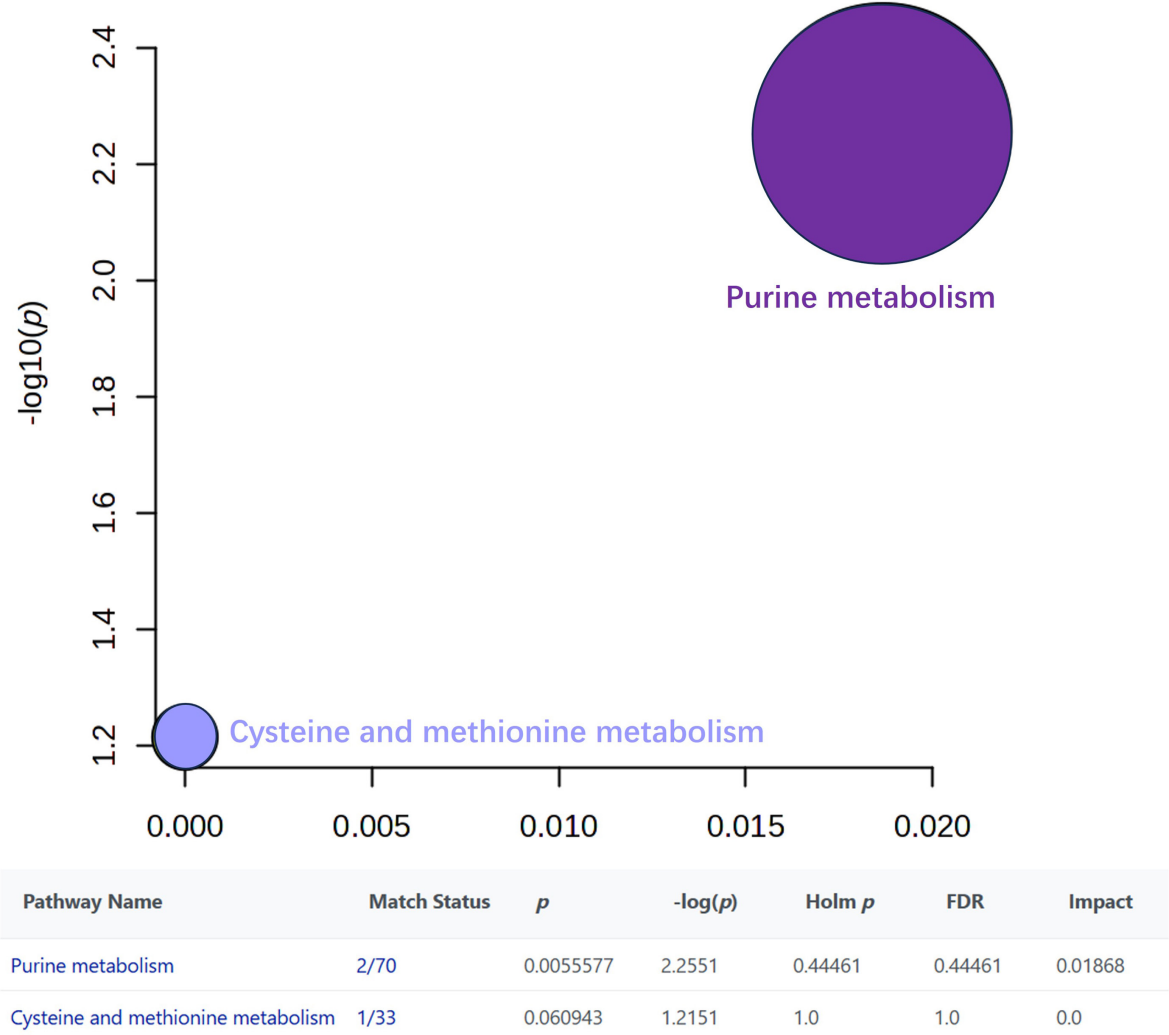
Overview of differential decreased metabolite pathway analysis. KEGG pathway enrichment analysis of the differentially downregulated metabolites indicated significant association with purine and cysteine‐methionine metabolism, with the purine metabolism showing stronger correlation.

Conversely, 12 metabolites were significantly downregulated in LCBM, primarily within the purine, cysteine, and methionine metabolism pathways (Figure 6). Purines and pyrimidines are essential for DNA replication and mRNA synthesis, directly influencing tumor cell proliferation and growth. Among them, pyrimidine nucleosides, such as uridine and deoxyuridine, can mitigate oxidative stress by reducing reactive oxygen species imbalance resulting from NADPH and glutathione depletion, thereby stabilizing the tumor microenvironment. In contrast, the purine nucleoside adenosine exerts immunosuppressive effects by inhibiting anti‐tumor immune responses. Consequently, dysregulation of purine and pyrimidine metabolism can profoundly affect tumor progression [18]. Lin Dongxin et al. demonstrated that aberrant purine metabolism in tumor cells is closely linked to the immune microenvironment, and deficiency in purine reductase may induce therapy resistance, offering new insights for immunotherapy [22].

The biological behavior of LCBM exhibits significant heterogeneity across different molecular subtypes, with EGFR‐mutant NSCLC demonstrating particularly strong neurotropism and therapeutic resistance [23, 24]. Our study reveals that these distinct clinical phenotypes stem from EGFR oncogene‐driven specific metabolic reprogramming, centrally characterized by the aberrant activation of phenylalanine and purine metabolic pathways. Specifically, sustained EGFR signaling activation not only upregulates amino acid transporters to facilitate phenylalanine uptake‐utilizing its metabolites to scavenge reactive oxygen species (ROS) and adapt to the oxidative stress of the brain microenvironment‐but also drives hyperactive purine synthesis. This provides essential nucleic acid precursors and key signaling molecules (e.g., GTP), establishing a “metabolism‐signaling” positive feedback loop that consolidates oncogenic drivers and mediates acquired drug resistance [25]. Furthermore, this process is intricately linked to monocarboxylate transporter (MCT)–mediated lactate shuttling and enhanced oxidative phosphorylation (OXPHOS), collectively constructing a survival defense line for tumor cells under targeted therapy pressure [26]. Consequently, the highly expressed metabolites identified in this study are not generalized indicators of tumor burden but rather represent a unique “metabolic fingerprint” driven by EGFR mutations. These markers not only signify a high‐risk propensity for brain metastasis but also profoundly imply potential resistance mechanisms to specific targeted therapies. This underscores the necessity for future validation of their specificity across distinct genotypic cohorts and warrants the exploration of combination intervention strategies targeting these metabolic nodes to overcome the bottlenecks of single‐agent targeted therapy.

In summary, this study successfully identified a panel of differentially expressed metabolites closely associated with LCBM, particularly those related to phenylalanine and purine metabolism. Evidence suggests that these metabolites are not merely indicators of tumor burden but also play profound roles in modulating the immune microenvironment and fostering therapeutic resistance, highlighting their potential as a “multidimensional composite biomarker panel.” Given the cross‐sectional design of the current study, its immediate clinical utility lies primarily in offering a novel molecular perspective for the noninvasive auxiliary diagnosis of LCBM, rather than serving as a direct substitute for established imaging standards. Specifically, this metabolic signature holds promise for supplementary application in two distinct scenarios: first, as an adjunctive tool for differential diagnosis in challenging cases where brain MRI findings are atypical, compromised by artifacts, or contraindicated due to patient intolerance to contrast enhancement, thereby providing additional molecular‐level evidence; and second, as an early warning signal to identify high‐risk subgroups among patients with newly diagnosed NSCLC, prompting clinicians to institute more rigorous neurological surveillance for these individuals. Nevertheless, translating these findings into routine clinical practice presents significant challenges. With the increasing accessibility of high‐resolution mass spectrometry and the optimization of multivariate data analysis methods, the primary objective for future research is to validate the robustness and generalizability of this model in independent prospective cohorts. Only through rigorous validation within clinical workflows can these metabolic biomarkers transition from laboratory discoveries to limited pilot clinical applications, ultimately providing a scientific basis for optimizing individualized management of LCBM.

## 4 Conclusions

In summary, through untargeted metabolomics analysis, this study successfully identified a panel of specific serum metabolic signatures closely associated with LCBM. Among the 32 screened differential metabolites, the upregulation of phenylalanine and arginine metabolism, coupled with the downregulation of purine and cysteine metabolism, delineates a unique metabolic reprogramming landscape in LCBM patients. This profile reflects the tumor's specialized requirements for energy supply, oxidative stress homeostasis, and immune modulation during adaptation to the brain microenvironment. These findings not only deepen our understanding of the metabolic mechanisms underlying LCBM “neurotropism” but also provide a list of noninvasive biomarkers with potential diagnostic value.

Given the cross‐sectional nature of this study, its primary contribution lies in offering a novel molecular perspective for the auxiliary diagnosis and initial risk screening of LCBM, rather than establishing a definitive clinical gold standard. Future efforts will prioritize validating the robustness and reproducibility of these 32 key metabolites in independent cohorts and preliminarily exploring their associations with specific driver genotypes (e.g., EGFR mutations). Although widespread routine clinical application remains a future goal, this study confirms the feasibility of serum metabolic profiling as a supplementary tool, laying a pragmatic foundation for developing early warning strategies for high‐risk populations and exploring potential metabolic intervention targets.

## 5 Limitations

While this study elucidates unique metabolic signatures associated with LCBM, several limitations must be considered when interpreting the findings.

First, constraints regarding study design and sample size: The single‐center, cross‐sectional design and the relatively modest sample size (*n* = 66) limit statistical power and increase the risk of model overfitting. Furthermore, recruitment from a single center may introduce spectrum bias, resulting in a lack of diversity in demographic characteristics, genetic backgrounds, and environmental exposures among the study population. This potentially compromises the generalizability of our conclusions to broader real‐world populations. Consequently, the identified metabolic biomarkers require further validation of their robustness in large‐scale, multicenter external cohorts.

Second, insufficient specificity of the control group: Although this study successfully differentiated LCBM patients from those with early‐stage lung cancer and healthy controls, it lacked a control group comprising Stage IV patients with extracranial metastases but without brain involvement. This omission makes it difficult to definitively determine whether the observed metabolic alterations are specific to cerebral involvement or merely reflect a high systemic tumor burden characteristic of advanced malignancy. Therefore, the reported metabolic features should currently be regarded as candidate biomarkers highly correlated with brain metastatic status; their specificity relative to noncentral nervous system metastatic diseases awaits direct comparative validation against a matched cohort with extracranial metastases.

Third, the depth of integration regarding molecular heterogeneity remains limited. Although we have supplemented the revised manuscript with driver gene status (e.g., EGFR and ALK) for the experimental group and observed that certain metabolites may exhibit higher specificity within the EGFR‐mutant subgroup, our sample size constraints precluded a comprehensive elucidation of the complex interplay between metabolic reprogramming and specific oncogenic signaling pathways. Future studies must expand the sample size to specifically enroll patients with diverse molecular subtypes, thereby clarifying whether these metabolic features serve as “pan‐cancer” warning signals for brain metastasis or merely as accompanying characteristics of specific molecular subtypes.

Finally, the maturity of the predictive model: The current research primarily focuses on the discovery and description of differential metabolites, representing a hypothesis‐generating phase. We have not yet constructed and validated a mature, artificial intelligence‐based independent clinical prediction model. The current metabolic “fingerprint” serves more as a qualitative “warning signal,” prompting clinicians to lower the threshold for contrast‐enhanced brain MRI in high‐risk patients (e.g., those with ambiguous neurological symptoms), rather than directly replacing existing diagnostic gold standards. Future work will aim to integrate longitudinal follow‐up data, utilizing receiver operating characteristic (ROC) curves and decision curve analysis (DCA) to determine optimal diagnostic cut‐off values and quantify the net benefit of this metabolism‐guided strategy in optimizing clinical resource allocation and improving patient outcomes.

## Author Contributions

Qi Wang and Xin You conceived and designed the study. Hongxia Zhu and Yinuo Jin performed the experiments and collected the data. Yinuo Jin analyzed the data and wrote the manuscript. All authors reviewed and approved the final manuscript.

## Funding

This work was supported by the Medical and Industry Joint Innovation Programs (DMU‐2 & DICP UN202302) from the Second Affiliated Hospital of Dalian Medical University and DICP; Liaoning Revitalization Talents Program (Grant/Award Number: XLYC2002013); the Liaoning Clinical Medical Research Center Construction Program; the “1+X” Program for Clinical Competency Enhancement‐Interdisciplinary Innovation Project of the Second Hospital of Dalian Medical University; and the Talent Innovation Support Plan of Dalian, China (No. 2021RQ008).

## Ethics Statement

The collection of serum samples used in this study was approved by the Ethics Committee of the Second Affiliated Hospital of Dalian Medical University.

## Conflicts of Interest

The authors declare no conflicts of interest.

## Supporting information




**Table S1:** Supplementary Information.

**Table S2:** Supplementary Information.

**Table S3:** Supplementary Information.

## Data Availability

The data that support the findings of this study are available in the Supporting Information of this article.
